# Polydopamine‐Integrated Porcine Small Intestine Decellularized Extracellular Matrix Hydrogel Microparticles for Wound Healing

**DOI:** 10.1002/smmd.70022

**Published:** 2025-11-18

**Authors:** Shangrui Rao, Hongzheng Li, Letian Meng, Lijun Cai, Weijian Sun, Yuyang Zhang

**Affiliations:** ^1^ Department of Colorectal and Anal Surgery The First Affiliated Hospital of Wenzhou Medical University Wenzhou China; ^2^ Department of Materials Science and Engineering College of Design and Engineering National University of Singapore Singapore Singapore; ^3^ Department of Gynecology The First Affiliated Hospital of Wenzhou Medical University Wenzhou China

**Keywords:** decellularized extracellular matrix, microparticles, polydopamine, porcine small intestine, wound healing

## Abstract

Decellularized materials show good prospects in wound healing. Current research focuses on developing advanced decellularized scaffolds and designing multifunctional structures to enhance tissue repair. In this study, a kind of polydopamine (PDA)‐integrated decellularized porcine small intestine tissue (dSI) and alginate (ALG) composite hydrogel microparticles (PDA@dSI/ALG) are proposed for efficient wound healing. These PDA@dSI/ALG are prepared via microfluidic electrospray technology combined with ionic crosslinking, followed by PDA coating. The dSI is derived from porcine small intestine tissue and is rich in various nutrients, which can promote the repair of damaged tissues. Moreover, incorporating PDA imparts the microparticles with superior photothermal responsiveness under near‐infrared irradiation, enabling efficient heat generation to eradicate bacteria. Overall, the PDA@dSI/ALG composite hydrogel microparticles combine extracellular matrix support with photothermal antibacterial function, underscoring their translational potential for future wound healing strategies.

## Key Points

- PDA@dSI/ALG hydrogel microparticles are fabricated via a simple microfluidic electrospray combined with ionic crosslinking.
- The dSI preserves structural proteins and bioactive factors from the extracellular matrix, including collagen and fibronectin, as well as various growth factors, which are crucial for tissue regeneration.
- PDA coating confers near‐infrared‐responsive photothermal antibacterial capability, ensuring effective bacterial eradication.
- The microparticles exhibit dual functionality by simultaneously supporting tissue repair and suppressing inflammation, while promoting angiogenesis and collagen deposition.

## Graphical abstract

Polydopamine‐coated microparticles derived from decellularized porcine small intestine combine the bioactivity of natural extracellular matrix with near‐infrared‐responsive antibacterial functionality, effectively enhancing wound healing.

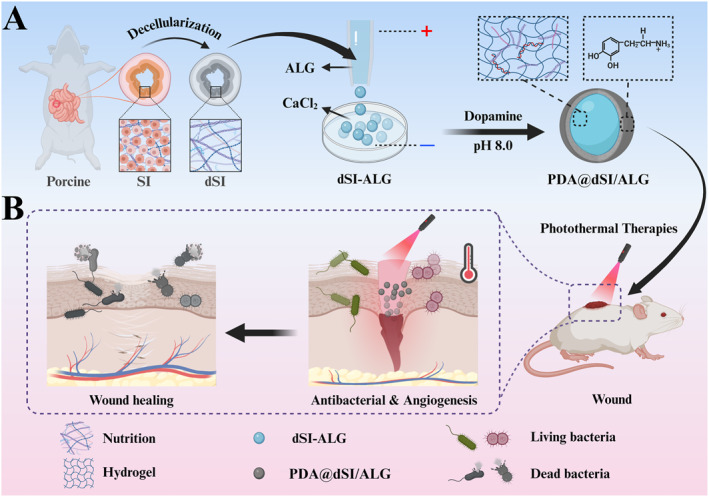

## 1 Introduction

As the primary protective organ of the human body, skin is prone to injury arising from diseases, trauma, or surgical interventions [1]. In response, increasing scientific attention has been directed toward the development of effective strategies for wound healing. Wound dressings, which are protective materials that promote healing, prevent infection, and manage wound moisture, have emerged as one promising method for wound healing because of notable therapeutic efficacy, ease of application, and good patient compliance [2, 3, 4]. To date, a variety of wound dressings have been developed for enhancing wound healing [5, 6, 7, 8]. Among them, nature‐derived wound dressings stand out due to their striking biocompatibility, biodegradability, and abundant availability [9, 10, 11, 12]. However, most natural dressings exhibit limited functionality and often require supplementation with growth factors or antibacterial agents to achieve comprehensive therapeutic effects [13, 14, 15, 16]. In addition, their complex processing procedures pose challenges for clinical translation [17, 18, 19]. Therefore, there is an urgent need to explore nature‐derived wound dressings with multifunction and facile preparation.

**FIGURE 1:**
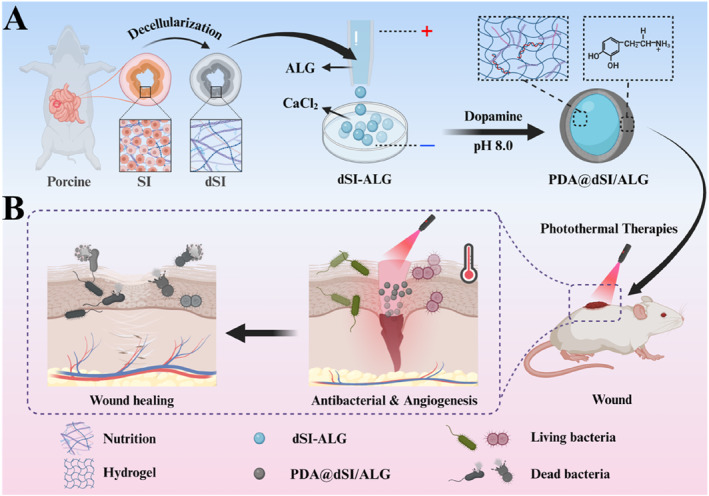
Schematic diagram showing the preparation and wound healing application of PDA@dSI/ALG microparticles. (A) Fabrication process of PDA@dSI/ALG microparticles. (B) Application of PDA@dSI/ALG microparticles for tissue repair.

This study reports the fabrication of hydrogel microparticles composed of decellularized porcine small intestine (dSI) and alginate (ALG), subsequently modified with polydopamine (PDA) and referred to as PDA@dSI/ALG, aiming to promote tissue repair, as illustrated in Figure 1. As a natural extracellular matrix scaffold rich in collagen, fibronectin, and growth factors, dSI contributes to regenerative processes and is considered a potential candidate in wound healing and tissue engineering [20, 21, 22, 23, 24]. Meanwhile, hydrogel microparticles are considered ideal platforms for wound healing due to their excellent adaptability to irregular wound geometries and their ability to support tissue regeneration [25, 26, 27]. By incorporating multifunctional materials such as PDA, the system can further enable intelligent and responsive wound healing strategies [28, 29, 30, 31]. Therefore, the integration of dSI with hydrogel microparticles offers a promising approach for developing high‐performance wound dressings.

Herein, we developed dSI‐based hydrogel microparticles for efficient wound healing using a one‐step microfluidic electrospray technique combined with ionic crosslinking. The dSI was obtained via nonionic detergent treatment, effectively removing cellular components while preserving extracellular matrix (ECM) nutrients essential for tissue regeneration. Subsequently, dSI was mixed with ALG to form an ionically crosslinkable pre‐gel solution. Through microfluidic electrospray, this pre‐gel was dispersed into uniform droplets and rapidly solidified into hydrogel microparticles upon contact with calcium ions (Ca^2+^) via ionic crosslinking. To enhance the functionality of the microparticles, a polydopamine (PDA) coating was applied to their surface, yielding PDA@dSI‐ALG microparticles. The PDA nanoparticles imparted excellent photothermal properties under near‐infrared (NIR) light irradiation, enabling localized heat generation for effective antibacterial action [28, 32]. These composite microparticles exhibited strong antibacterial activity, favorable biocompatibility, and abundant bioactive components, collectively contributing to enhanced wound healing. By employing a wound model of mice, we proved that the PDA@dSI‐ALG microparticles could significantly accelerate wound closure by promoting bacterial clearance, reducing inflammatory response and stimulating angiogenesis.

## 2 Results and Discussion

**FIGURE 2:**
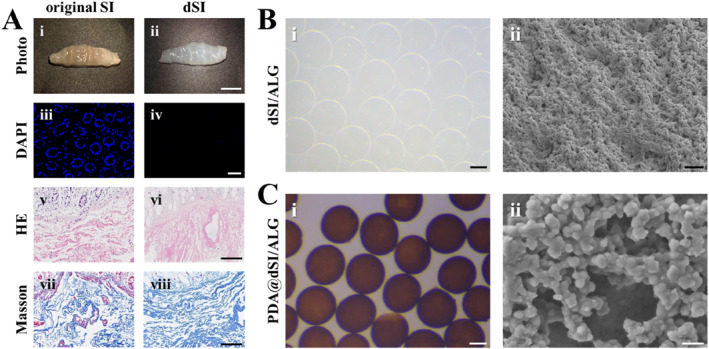
Preparation and characterization of PDA@dSI/ALG microparticles. (A) Representative optical, DAPI, HE and Masson images of original SI and dSI. (B) dSI‐ALG microparticles imaged by optical microscopy (i) and scanning electron microscopy (ii). (C) PDA@dSI/ALG microparticles imaged by optical microscopy (i) and scanning electron microscopy (ii). Scale bars: 0.5 cm for A(i and ii); 50 μm for A(iii and iv); 100 μm for A(v–viii), B(i), C(i); 2 μm for B(ii); 0.5 μm for C(ii).

Under representative experimental conditions, we chose decellularized porcine small intestine tissue (dSI) as the base material. Specifically, the non‐ionic surfactant Triton X‐100 removed nuclear components from dSI while preserving its bioactive constituents [33, 34]. As shown in Figure 2A, the original porcine small intestine (SI) tissue was light pink, while after decellularization, the dSI showed a transparent gelatinous morphology. Decellularization was assessed by 4′,6‐diamidino‐2‐phenylindole (DAPI) in combination with hematoxylin‐eosin (H&E) and Masson's trichrome staining [34]. DAPI analysis showed no observable nuclei in the dSI tissue, demonstrating that intestinal cells were thoroughly eliminated (Figure 2A iii and iv). HE and Masson staining indicated that essential extracellular matrix components, particularly fibronectin and collagen, were well preserved following the decellularization process, which are critical for supporting tissue regeneration (Figure 2A v–viii). In addition, DNA quantification showed that the residual dsDNA content after decellularization was below 50 ng/mg (Supporting Information S1: Figure S1), complying with the safety threshold for decellularized materials and supporting the low immunogenicity of the dSI matrix. It's noteworthy that pepsin was introduced for further digestion to enhance the availability of dSI.

To fabricate dSI‐based microparticles, we mixed the dSI with alginate (ALG) to form an ionically crosslinkable pre‐gel solution. Then, the pre‐gel was fabricated into microparticles via microfluidic electrospray based on the constructed microfluidic device (Supporting Information S1: Figure S2). Specifically, the high voltage of microfluidic electrospray can drive the continuous pre‐gel into dispersed droplets. The droplets were immediately collected in a calcium chloride (CaCl_2_) solution where they went through ionic crosslinking, forming dSI‐ALG hydrogel microparticles with a transparent appearance (Figure 2B i). The morphology of the resultant microparticles was characterized by SEM (Figure 2B ii and Supporting Information S1: Figure S3). Results showed that dSI‐ALG microparticles were spherical and had a porous surface. In addition, the dSI‐ALG particles also showed good dispersibility, with an average particle size of about 200 μm (Supporting Information S1: Figure S4A). To broaden the functionality of the microparticles, we coated the dSI‐ALG particles with polydopamine (PDA). Immersion of dSI‐ALG microparticles in an alkaline dopamine solution led to PDA coating formation through dopamine self‐polymerization, resulting in PDA@dSI/ALG microparticles (Figure 2C i). The PDA@dSI/ALG microparticles appeared black in appearance and SEM showed that PDA was successfully coated on the surface of the microparticles (Figure 2C ii). The PDA@dSI/ALG microparticles also showed good dispersibility, and their particle size was similar to that of the dSI‐ALG microparticles (Supporting Information S1: Figure S4B), indicating that the coating process of PDA had little influence on the size of these microparticles.

**FIGURE 3:**
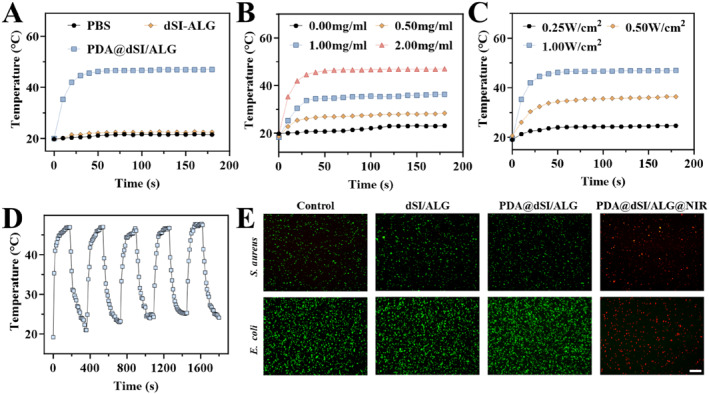
Photothermal response of PDA@dSI/ALG microparticles. (A) Thermal response of PBS, dSI–ALG, and PDA@dSI/ALG microparticles under NIR irradiation (PDA@dSI/ALG with 2.0 mg/mL PDA was tested under laser irradiation at 1.00 W/cm^2^). (B) Microparticles with different PDA coating levels were monitored for thermal response during NIR irradiation at 0.75 W/cm^‐2^. (C) Thermal response of 2.0 mg/mL PDA@dSI/ALG microparticles under varying laser power densities. (D) Recorded temperature trend of PDA@dSI/ALG microparticles over five repeated NIR on/off cycles. (E) Live/dead staining images of *S. aureus* and *E. coli* under different treatments. Scale bars: 200 μm for E.

The integration of PDA endowed the PDA@dSI/ALG microparticles with good photothermal conversion capabilities. To verify their performance, temperature changes were recorded under 808 nm near‐infrared (NIR) light. It was observed that the temperature of the PDA@dSI‐ALG increased significantly after NIR irradiation, while PBS and dSI‐ALG microparticles showed little temperature variation, confirming that PDA imparted the microparticles with photothermal response property (Figure 3A). In further research, we found that the temperature of the PDA@dSI/ALG microparticles shows a significant upward trend with the increasing of laser power density and PDA concentration (Figure 3B,C). For example, under 1.0 W/cm^2^ laser irradiation, the temperature of PDA@dSI/ALG microparticles coated with a PDA concentration of 2.0 mg/mL can rise to about 48°C. In addition, the microparticles could be repeatedly heated in 5 laser switching cycles, verifying their good photothermal stability (Figure 3D). To achieve effective photothermal antibacterial performance without causing thermal damage to skin tissue, subsequent experiments were carried out using 1.0 W/cm^2^ laser irradiation and PDA@dSI/ALG particles containing 2.0 mg/mL of PDA.

Benefiting from their photothermal response, PDA@dSI/ALG microparticles generate heat that causes protein denaturation and damages bacterial membranes, ultimately achieving efficient sterilization. To verify this, the materials were co‐cultured with *S. aureus* and *E. coli*. It was found that only PDA@dSI/ALG exhibited significant antibacterial effects under NIR irradiation, with red (dead bacteria) being the main color in the fluorescence image and green (surviving bacteria) being very rare (Figure 3E). In contrast, the other groups were mainly green fluorescent, indicating that the bacteria survived in large numbers. Quantitative analysis further confirmed this phenomenon (Supporting Information S1: Figure S5). Under NIR irradiation, the PDA@dSI/ALG group showed survival rates of approximately 4% for *S. aureus* and 2% for *E. coli*. These results demonstrate that the PDA@dSI/ALG microparticles possess photothermal antibacterial ability under near‐infrared excitation.

**FIGURE 4:**
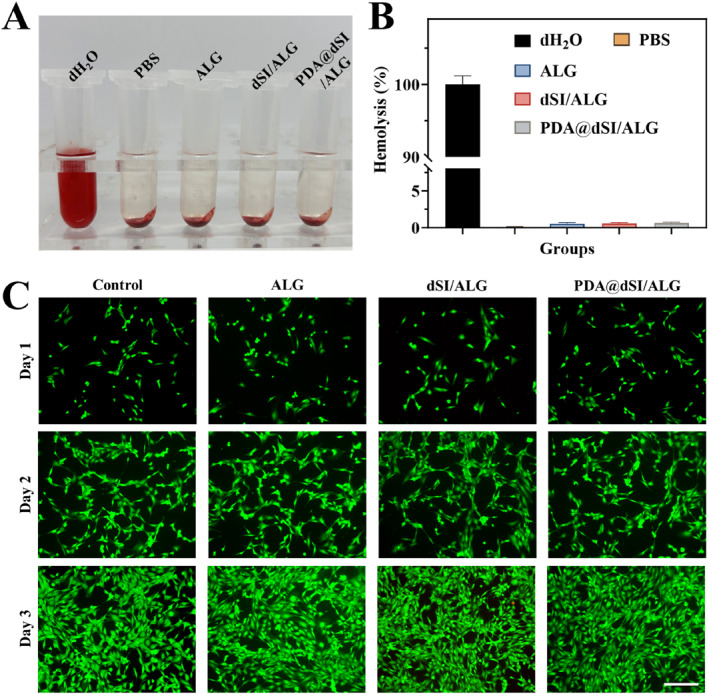
Safety performance testing of PDA@dSI/ALG microparticles. (A) Representative photographs of hemolysis assay solutions. (B) Corresponding hemolysis percentages of different groups. (C) Representative live/dead staining fluorescence images of different groups. Scale bars: 200 μm for C.

Given their direct interaction with blood and tissue, wound dressings must possess excellent hemocompatibility and biocompatibility to ensure safe and effective application. We then carried out hemolysis test to cell coculture to evaluate the biosafety of PDA@dSI/ALG microparticles. As can be seen in Figure 4A, the dH_2_O control exhibited a strong red coloration, indicating severe hemolysis, while the solutions of the PBS, ALG, dSI‐ALG, and PDA@dSI/ALG groups were almost transparent. The hemolysis rate results showed that except for the positive group, the hemolysis rates of the other groups were all lower than 5% (Figure 4B), which met the blood compatibility standards. Furthermore, we co‐cultured the microparticle extract with NIH‐3T3 cells to evaluate its biosafety. The live/dead assay results indicated that all groups supported robust cell proliferation, reflecting good biocompatibility (Figure 4C). CCK‐8 analysis revealed stability in cell viability, aligning with the previous observations (Supporting Information S1: Figure S6). In summary, PDA@dSI/ALG microparticles have good biocompatibility and blood compatibility and are suitable for subsequent in vivo wound repair studies.

**FIGURE 5:**
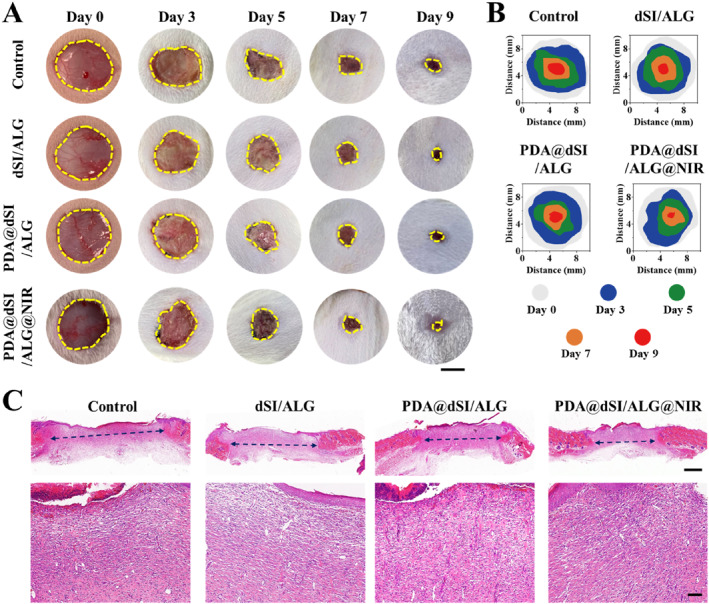
Progress of wound repair in the skin defect model. (A) Images of dorsal wounds in rats subjected to different treatments, together with quantitative analysis of wound area. (B) Wound area tracking analysis corresponds to the photograph shown in (A). (C) Granulation tissue width on day 9 evaluated by H&E staining. Scale bars: 0.5 cm for A, 1.5 mm for C; 100 μm for C (magnified view).

PDA@dSI/ALG microparticles were tested for their healing capacity in a rat dorsal skin wound model. Subsequently, the animals were randomly allocated to four groups: PBS control, dSI–ALG, non‐irradiated PDA@dSI/ALG, and PDA@dSI/ALG@NIR under NIR irradiation. During treatment, thermal imaging (Supporting Information S1: Figure S7) was used to monitor the temperature in the PDA@dSI/ALG@NIR group. The results indicated that after NIR exposure, the‐site temperature reached about 50°C in 1 minute and stabilized, effectively initiating and sustaining the antibacterial effect. The extent of wound closure was assessed at sequential postoperative days 0,3,5,7 and 9 (Figure 5A). According to the results, wound repair in the PDA@dSI/ALG combined with NIR treatment was significantly enhanced when contrasted with the remaining groups. This phenomenon is likely associated with a PDA layer covering the microparticle surface, which confers NIR‐triggered antibacterial capability (Figure 5B). Further observation of tissue repair by H&E staining showed that new granulation tissue was generated in all groups, but the PDA@dSI/ALG@NIR group was the most obvious, with the smallest wound width (Figure 5C and Supporting Information S1: Figure S8). In summary, PDA@dSI/ALG microparticles can promote the wound healing process benefiting from their NIR‐responsive antibacterial abilities, preliminarily verifying their NIR‐dependent therapeutic potential.

**FIGURE 6:**
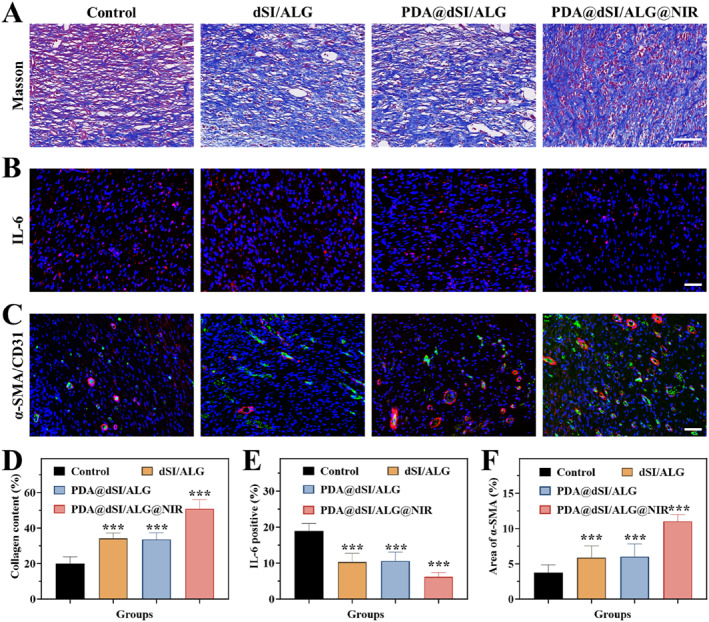
Evaluation of collagen accumulation, vascular formation, and inflammation in wound sites. (A–C) Histological and immunofluorescent images of wound tissue: (A) Masson's trichrome for collagen fibers; (B) IL‐6 (red); (C) CD31 (red) together with *α*‐SMA (green). (D–F) Quantifications of (D) collagen, (E) IL‐6, and (F) CD31. Scale bars: 100 μm for A; 50 μm for B and C. Data are presented as means ± SD (*n* = 5). ***p < 0.001 compared with the control group.

To further assess tissue remodeling, particular attention was given to collagen deposition. Masson staining was carried out to observe collagen deposition. The PDA@dSI/ALG@NIR group showed the greatest collagen deposition, suggesting an accelerated healing process (Figure 6A and D). On day 9, immunohistochemical staining assessed interleukin‐6 (IL‐6) levels. Relative to the control group, which exhibited marked inflammation, IL‐6 expression in the PDA@dSI/ALG@NIR group was markedly lower, suggesting strong anti‐inflammatory activity and low immunogenic profile of the material (Figure 6B and E). Angiogenesis was assessed by detecting *α*‐SMA (smooth muscle marker) and CD31 (endothelial marker). Analysis revealed that neovascularization was limited in the control group, accompanied by low vascular marker expression, whereas PDA@dSI/ALG combined with NIR significantly upregulated these markers, resulting in enhanced angiogenesis and the formation of more mature vascular structures (Figure 6C and F). Based on the above results, PDA@dSI/ALG microparticles promoted tissue regeneration, reduced inflammation, and enhanced angiogenesis without causing thermal or immune‐related damage, indicating good potential for skin repair.

## 3 Conclusion and Discussion

In summary, this study developed natural functional PDA@dSI/ALG microparticles for effective wound treatment. The microparticles retain the extracellular matrix components of porcine small intestinal tissue, including structural proteins, cytokines, and growth factors, which facilitate regeneration and create a favorable milieu for tissue healing. In addition, the introduction of PDA gave the microparticles near‐infrared induced photothermal antibacterial properties. These features not only help suppress inflammation in the wound area but also promote faster tissue repair and regeneration. The in vivo results demonstrated not only enhanced wound closure but also reduced inflammatory markers, highlighting their therapeutic potential. While promising, this strategy still faces limitations, including batch‐to‐batch variability of biological materials, preparation standardization, and concerns about long‐term storage stability. Nevertheless, their natural origins, multifunctional properties, and ease of topical application suggest strong potential for future clinical translation in wound care.

## 4 Experimental Section

Reagents and Animals: The primary reagents included ALG and CaCl_2_ from Sigma‐Aldrich (USA), along with dopamine hydrochloride sourced from MacLean Reagents (China). Pepsin, glacial acetic acid, and Triton X‐100 were analytical grade chemicals. SYTO/PI fluorescent dyes and DNA extraction kits (KeyGEN BioTECH, Jiangsu, China) together with DAPI dye (Solarbio, Beijing, China) were used for staining and nucleic acid assays. Antibodies against IL‐6 and *α*‐SMA (Biosun Biotech, Wuhan, China) and CD31 antibody (Abcam, UK) were applied in immunostaining experiments. For characterization, optical imaging was performed with an Olympus inverted microscope, fluorescence observation (live/dead staining) with an Axio Vert A1 system, morphological analysis with a Hitachi SU8010 scanning electron microscope (SEM), and infrared thermal imaging with a FLIR E5‐XT device (Germany).

Preparation of dSI: Fresh porcine small intestine was cut into pieces of approximately 0.5 cm and thoroughly washed with PBS containing antibiotics to remove blood and impurities. The cleaned tissue was then placed in a mixture of 1% Triton X‐100% and 0.1% ammonia and gently shaken at room temperature for 48 h, with the solution changed regularly to ensure effective decellularization. After treatment, the sample was freeze‐dried and ground into a powder. The resulting powder was then added to a digestive solution composed of 1% pepsin and 100 mM acetic acid and digested for 48 h, ultimately forming a uniform dSI solution for the subsequent construction of functional hydrogel microparticles.

DNA content detection: To evaluate the decellularization effect, DAPI staining was first performed on tissue samples before and after decellularization. Fluorescence microscopy was used to observe the residual cell nucleus to determine whether the nuclear components were effectively removed. Subsequently, total DNA from tissue samples was extracted using a commercial DNA extraction kit (KeyGEN BioTECH, Jiangsu, China). After extraction, the absorbance of DNA was measured using an ultraviolet spectrophotometer and its concentration was calculated. Results were normalized to tissue dry weight, and DNA content was expressed in nanograms per milligram (ng/mg).

Preparation of PDA@dSI‐ALG microparticles: 2wt% dSI solution was thoroughly mixed with 1.5wt% ALG to prepare a pre‐gel solution. Using a microfluidic electrospray device, the pre‐gel solution is atomized into tiny droplets under the action of high voltage and dripped into a pre‐configured CaCl_2_ solution. Stable dSI/ALG hydrogel microparticles were formed through ionic crosslinking. The obtained microparticles were collected and further placed in an alkaline dopamine solution and soaked overnight. Dopamine self‐polymerizes and coats the surface of the microparticles to form a polydopamine (PDA) coating. Finally, we obtained surface functionalized PDA@dSI/ALG composite particles.

Photothermal performance test: The PDA@dSI/ALG hydrogel microparticles were placed under a near‐infrared laser to test their photothermal properties. A light source with a wavelength of 808 nm was used for irradiation, with the tip positioned 10 cm above the sample surface, and the temperature changes of the particles under different conditions were recorded in real time. By setting different laser power densities and PDA concentrations, the corresponding temperature rise effects were tested to evaluate the photothermal response performance. To further verify its photothermal stability, five rounds of laser switching cyclic heating experiments were carried out, and the temperature change curves during each round of heating and cooling were recorded.

Antibacterial performance test: PDA@dSI/ALG hydrogel microparticles were tested against *S. aureus* and separately against *E. coli*, with each bacterial suspension maintained in contact for 4 h. Samples underwent 10 min of near‐infrared exposure (808 nm, 1.0 W/cm^2^), with the tip positioned 10 cm above the sample surface. After treatment, the bacterial viability was examined through fluorescence observation. By comparing the fluorescence images of different experimental groups and using image analysis software to quantitatively calculate the bacterial survival rate, the antibacterial effect of the particles under near‐infrared irradiation conditions was evaluated.

Blood compatibility testing: The hemocompatibility of PDA@dSI/ALG microparticles was assessed through a hemolysis assay. Fresh anticoagulated mouse blood was combined with various test samples (PBS, ALG, dSI/ALG, and PDA@dSI/ALG) and incubated in a 37°C water bath, with distilled water serving as the positive control. After 3 h of incubation, the supernatant obtained by centrifugation was analyzed for absorbance to calculate hemolysis percentage. These values were then used to judge the safety of the microparticles during blood contact.

Biocompatibility assessment: To evaluate the biosafety of PDA@dSI/ALG microparticles, pre‐prepared extracts were co‐cultured with NIH‐3T3 fibroblasts. After one, two, and 3 days of co‐culture, cells were stained for live/dead status, and cell morphology and viability were observed using fluorescence microscopy. Cell proliferation activity was assessed using CCK‐8 assays.

In vivo evaluation of healing performance: To investigate the regenerative potential of PDA@dSI/ALG microparticles, a 1 cm full‐thickness skin injury model was created on the dorsal region of Sprague‐Dawley rats. The animals were randomly assigned to four experimental conditions: control (PBS‐treated), dSI/ALG, PDA@dSI/ALG, and PDA@dSI/ALG combined with NIR irradiation. In the latter, 808 nm laser exposure (1.0 W/cm^2^, 10 min) was applied during therapy. Wound status was documented on postoperative days 0,3,5,7 and 9. On the ninth day, excised wound samples underwent histological examination with H&E and Masson's methods to evaluate structural restoration and extracellular matrix organization. Inflammatory activity was characterized through IL‐6–specific immunolabeling, while neovascularization was examined by simultaneous detection of CD31 and *α*‐SMA in the same tissue sections. Sprague‐Dawley rats (200–250g) were utilized in the experiments. (*n* = 5 for each group).

## Author Contributions

Y.Z. conceptualized and designed the study. S.R. performed the experiments. H.L. and L.M. provided support in data analysis and manuscript writing. L.C., W.S., and Y.Z. participated in scientific discussions and contributed to manuscript revision. S.R. and H.L. contributed equally to this work.

## Ethics Statement

All experiments involving rats were conducted in accordance with institutional guidelines for animal care and use. The Sprague‐Dawley rats were obtained from Zhejiang Charles River Laboratory Animal Technology Co., Ltd., and all procedures were approved by the Animal Ethics Committee of Wenzhou Institute of University of Chinese Academy of Sciences (Approval No. WIUCAS23071303).

## Conflicts of Interest

The authors declare no conflicts of interest.

## Supporting information


Supporting Information S1

## Data Availability

The data that support the findings of this study are available from the corresponding author upon reasonable request.

## References

[1] B. K. Nahak , J. R. Chowdhury , M. K. Sharma , et al., “Advancements in Multimodal Approaches for Enhanced Wound Healing: From Chemical to Physical Strategies,” Materials Today 88 (2025): 1087–1125.

[2] F. Mehvari , V. Ramezanzade , J. An , J. Kim , M. Dinari , and J. S. Kim , “Biopolymer‐Based Hydrogels for Biomedical Applications: Bioactivity and Wound Healing Properties,” Coordination Chemistry Reviews 518 (2024): 216093.

[3] B. Guo , R. Dong , Y. Liang , and M. Li , “Haemostatic Materials for Wound Healing Applications,” Nature Reviews Chemistry 5 (2021): 773–791. 37117664 10.1038/s41570-021-00323-z

[4] R. Yu , H. Zhang , and B. Guo , “Conductive Biomaterials as Bioactive Wound Dressing for Wound Healing and Skin Tissue Engineering,” Nano‐Micro Letters 14 (2022): 1. 10.1007/s40820-021-00751-y PMC8639891 34859323

[5] Y. Liang , J. He , and B. Guo , “Functional Hydrogels as Wound Dressing to Enhance Wound Healing,” ACS Nano 15 (2021): 12687–12722. 34374515 10.1021/acsnano.1c04206

[6] L. Zhang , Y. Yang , J. Wang , H. Zhang , Z. Zhang , and B. Guo , “A Portable, Sprayable, Highly Malleable, Elastic, and Hydrophobic Antibacterial Fibrous Wound Dressing for Infected Wound Healing,” Advanced Fiber Materials 7 (2025): 528–540.

[7] N. Tang , R. Zhang , Y. Zheng , et al., “Highly Efficient Self‐Healing Multifunctional Dressing With Antibacterial Activity for Sutureless Wound Closure and Infected Wound Monitoring,” Advanced Materials 34 (2022): 2106842. 10.1002/adma.202106842 34741350

[8] L. Fan , L. Wang , X. Wang , M. Li , H. Gu , and H. Zhang , “Multifunctional Silk and Gelatin Composed Microneedle Patches for Enhanced Wound Healing,” Smart Medicine 4 (2025): e137. 40059965 10.1002/smmd.137 PMC11862109

[9] H. Baniasadi , “State‐Of‐The‐Art in Natural Hydrogel‐Based Wound Dressings: Design, Functionalization, and Fabrication Approaches,” Advances in Colloid and Interface Science 342 (2025): 103527. 40300490 10.1016/j.cis.2025.103527

[10] Z. Huang , H. An , H. Guo , et al., “An Asymmetric Natural Nanofiber With Rapid Temperature Responsive Detachability Inspired by Andrias davidianus for Full‐Thickness Skin Wound Healing,” Advanced Fiber Materials 6 (2024): 473–488.

[11] M. Kharaziha , T. Scheibel , and S. Salehi , “Multifunctional Naturally Derived Bioadhesives: From Strategic Molecular Design Toward Advanced Biomedical Applications,” Progress in Polymer Science 150 (2024): 101792.

[12] H. Li , X. Lin , S. Rao , et al., “Decellularized Tumor Tissues Integrated With Polydopamine for Wound Healing,” Research 7 (2024): 0445 39109247 10.34133/research.0445 PMC11301524

[13] O. Yazarlu , M. Iranshahi , H. R. K. Kashani , et al., “Perspective on the Application of Medicinal Plants and Natural Products in Wound Healing: A Mechanistic Review,” Pharmacological Research 174 (2021): 105841. 34419563 10.1016/j.phrs.2021.105841

[14] Z. Liu , X. Liu , L. Bao , et al., “The Evaluation of Functional Small Intestinal Submucosa for Abdominal Wall Defect Repair in a Rat Model: Potent Effect of Sequential Release of VEGF and TGF‐β1 on Host Integration,” Biomaterials 276 (2021): 120999. 34273685 10.1016/j.biomaterials.2021.120999

[15] J. E. Allen , “IL‐4 and IL‐13: Regulators and Effectors of Wound Repair,” Annual Review of Immunology 41 (2023): 229–254. 10.1146/annurev-immunol-101921-041206 36737597

[16] X. Chen , L. Ren , H. Zhang , et al., “Basic Fibroblast Growth Factor‐Loaded Methacrylate Gelatin Hydrogel Microspheres for Spinal Nerve Regeneration,” Smart Medicine 2 (2023): e20220038. 39188281 10.1002/SMMD.20220038 PMC11235853

[17] N. Eslahi , F. Soleimani , R. Lotfi , F. Mohandes , A. Simchi , and M. Razavi , “How Biomimetic Nanofibers Advance the Realm of Cutaneous Wound Management: The State‐of‐the‐Art and Future Prospects,” Progress in Materials Science 145 (2024): 101293.

[18] Q. Li , X. Lai , Y. Duan , et al., “3D Nanofiber Sponge Based on Natural Insect Quaternized Chitosan/Pullulan/Citric Acid for Accelerating Wound Healing,” Carbohydrate Polymers 348 (2025): 122827. 39562102 10.1016/j.carbpol.2024.122827

[19] W. Zhang , L. Cai , J. Gan , and Y. Zhao , “Photothermal Responsive Porous Hollow Microneedles as Chinese Medicine Versatile Delivery System for Wound Healing,” Smart Medicine 3 (2024): e20240007. 39420949 10.1002/SMMD.20240007 PMC11425051

[20] Y. T. Song , Y. Q. Li , M. X. Tian , et al., “Application of antibody‐conjugated Small Intestine Submucosa to Capture Urine‐Derived Stem Cells for Bladder Repair in a Rabbit Model,” Bioactive Materials 14 (2022): 443–455. 35415280 10.1016/j.bioactmat.2021.11.017 PMC8978277

[21] J. Tan , Q. Y. Zhang , Y. T. Song , et al., “Accelerated Bone Defect Regeneration Through Sequential Activation of the M1 and M2 Phenotypes of Macrophages by a Composite BMP‐2@SIS Hydrogel: An Immunomodulatory Perspective,” Composites Part B: Engineering 243 (2022): 110149.

[22] X. Z. Zhang , Y. L. Jiang , J. G. Hu , et al., “Procyanidins‐Crosslinked Small Intestine Submucosa: A Bladder Patch Promotes Smooth Muscle Regeneration and Bladder Function Restoration in a Rabbit Model,” Bioactive Materials 6 (2021): 1827–1838. 33336114 10.1016/j.bioactmat.2020.11.023 PMC7721664

[23] H. Ren , D. Huang , M. Qiu , et al., “Microfluidic 3D Printing Hydrogels Based on Fish Liver Decellularized Extracellular Matrix for Liver Regeneration,” Smart Medicine 3 (2024): e20240056. 39776591 10.1002/SMMD.20240056 PMC11669779

[24] V. T. Duong , H. D. Nguyen , N. H. Luong , C. Chang , and C. Lin , “Photo‐Responsive Decellularized Small Intestine Submucosa Hydrogels,” Advanced Functional Materials 34 (2024): 2401952. 39525288 10.1002/adfm.202401952 PMC11546089

[25] L. Wang , X. Ding , L. Fan , et al., “Self‐Healing Dynamic Hydrogel Microparticles With Structural Color for Wound Management,” Nano‐Micro Letters 16 (2024): 232. 38954118 10.1007/s40820-024-01422-4 PMC11219637

[26] Q. Li , B. Chang , H. Dong , and X. Liu , “Functional Microspheres for Tissue Regeneration,” Bioactive Materials 25 (2023): 485–499. 37056261 10.1016/j.bioactmat.2022.07.025 PMC10087113

[27] D. Liang , G. Kuang , X. Chen , J. Lu , L. Shang , and W. Sun , “Near‐Infrared Light‐Responsive Nitric Oxide Microcarrier for Multimodal Tumor Therapy,” Smart Medicine 2 (2023): e20230016. 39188343 10.1002/SMMD.20230016 PMC11236066

[28] Y. Xu , J. Hu , J. Hu , et al., “Bioinspired Polydopamine Hydrogels: Strategies and Applications,” Progress in Polymer Science 146 (2023): 101740.

[29] H. Xu , Y. Zhang , H. Zhang , et al., “Smart Polydopamine‐Based Nanoplatforms for Biomedical Applications: State‐of‐Art and Further Perspectives,” Coordination Chemistry Reviews 488 (2023): 215153.

[30] L. Jin , X. Guo , D. Gao , et al., “An NIR Photothermal‐Responsive Hybrid Hydrogel for Enhanced Wound Healing,” Bioactive Materials 16 (2022): 162–172. 35415283 10.1016/j.bioactmat.2022.03.006 PMC8965777

[31] Y. Wang , Y. Zhang , Y. P. Yang , et al., “Versatile Dopamine‐Functionalized Hyaluronic Acid‐Recombinant Human Collagen Hydrogel Promoting Diabetic Wound Healing via Inflammation Control and Vascularization Tissue Regeneration,” Bioactive Materials 35 (2024): 330–345. 38379700 10.1016/j.bioactmat.2024.02.010 PMC10876488

[32] J. Kong , S. Ma , R. Chu , et al., “Photothermal and Photocatalytic Glycol Chitosan and Polydopamine‐Grafted Oxygen Vacancy Bismuth Oxyiodide (BiO_1‐X_I) Nanoparticles for the Diagnosis and Targeted Therapy of Diabetic Wounds,” Advanced Materials 36 (2024): 2307695. 10.1002/adma.202307695 38150667

[33] A. A. Golebiowska , J. T. Intravaia , V. M. Sathe , S. G. Kumbar , and S. P. Nukavarapu , “Decellularized Extracellular Matrix Biomaterials for Regenerative Therapies: Advances, Challenges and Clinical Prospects,” Bioactive Materials 32 (2024): 98–123. 37927899 10.1016/j.bioactmat.2023.09.017 PMC10622743

[34] B. S. Moura , M. V. Monteiro , L. P. Ferreira , P. Lavrador , V. M. Gaspar , and J. F. Mano , “Advancing Tissue Decellularized Hydrogels for Engineering Human Organoids,” Advanced Functional Materials 32 (2022): 2202825.

