# Supplementary material for: Polydopamine‐Integrated Porcine Small Intestine Decellularized Extracellular Matrix Hydrogel Microparticles for Wound Healing
Source: Smart Med. 2025 Nov 18;4(4):e70022. doi: 10.1002/smmd.70022 (PMC12631813; doi:10.1002/smmd.70022)
Supplement: Supplementary file 1 — Supporting Information S1 [file SMMD-4-e70022-s001.docx]

Supporting Information

**Polydopamine-integrated porcine small intestine decellularized extracellular matrix hydrogel microparticles for wound healing**

Shangrui Rao^#^, Hongzheng Li^#^, Letian Meng, Lijun Cai*, Weijian Sun*, Yuyang Zhang*


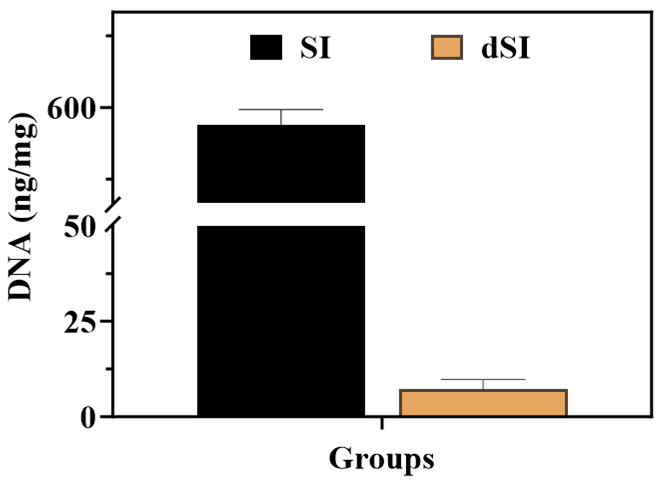


**Figure S1.** DNA quantification of SI and dSI.


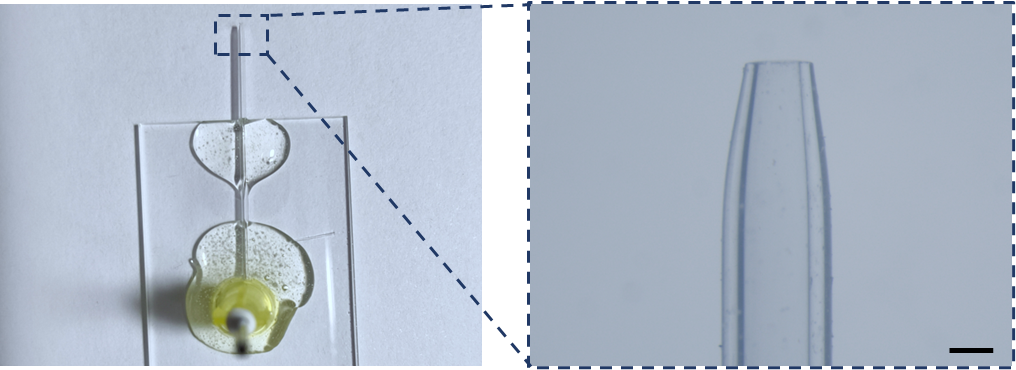


**Figure S2.** Optical image of the microfluidic device. Scale bars: 350 μm.


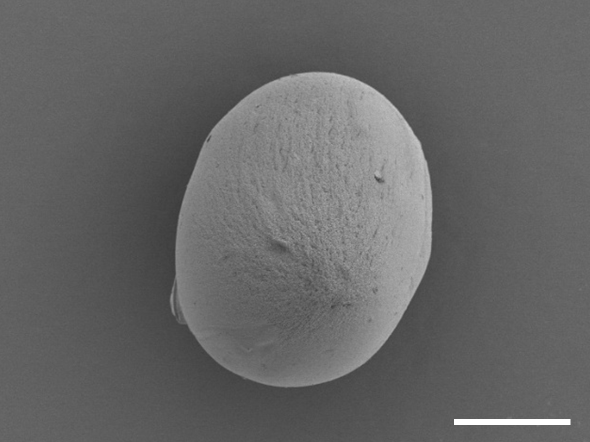


**Figure S3.** SEM image of dSI-ALG microparticles. Scale bars: 100 μm.


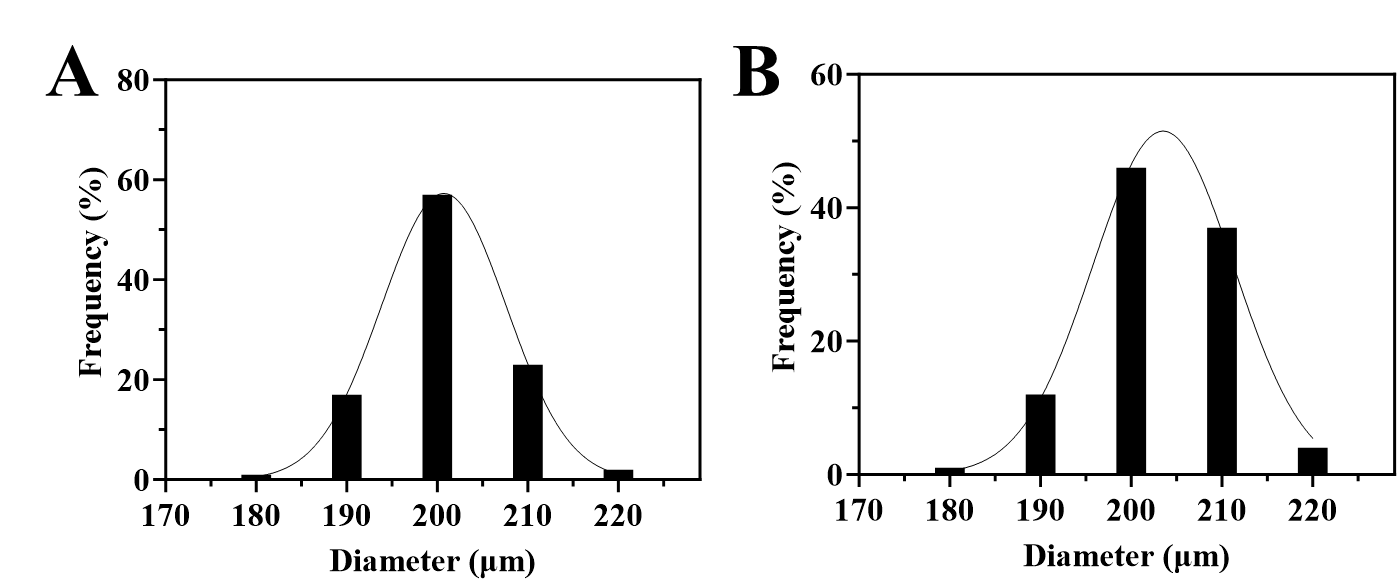


**Figure S4.** Diameter distribution of dSI-ALG (A) and PDA@dSI/ALG (B) microparticles.


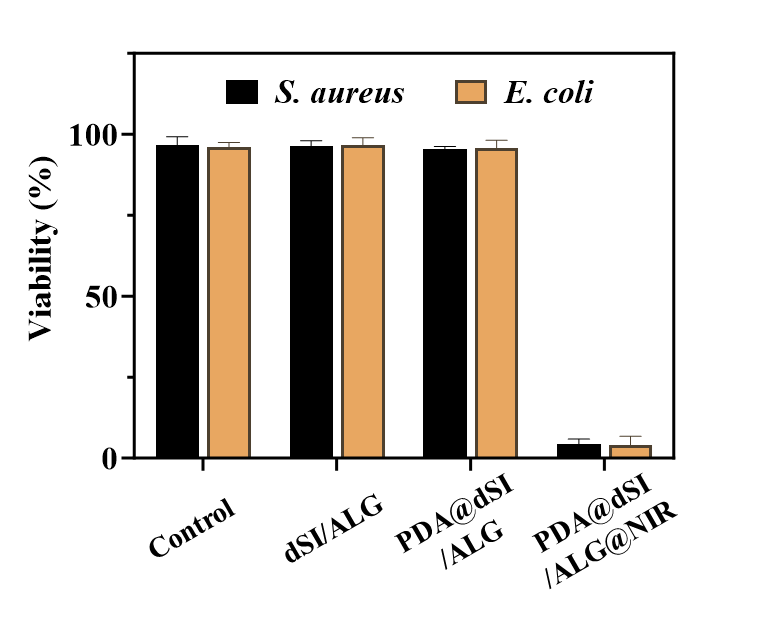


**Figure S5.** Quantitative evaluation of *E. coli* and *S. aureus* survival following various treatment protocols.


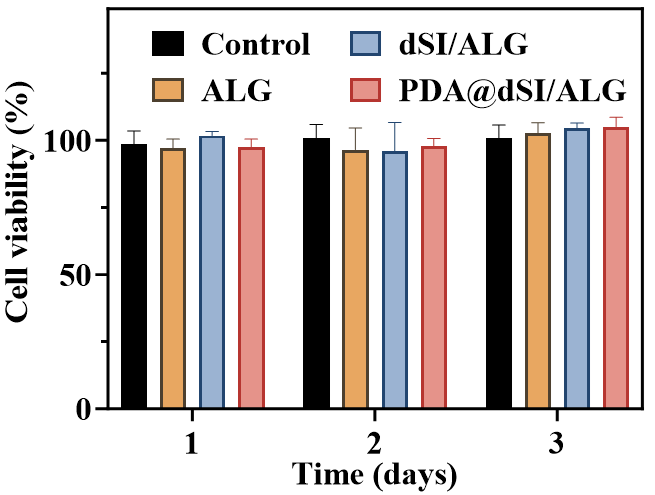


**Figure S6.** Survival rates of NIH-3T3 cells subjected to different interventions.

**Figure S7.** PDA@dSI/ALG for temperature recording during wound treatment.


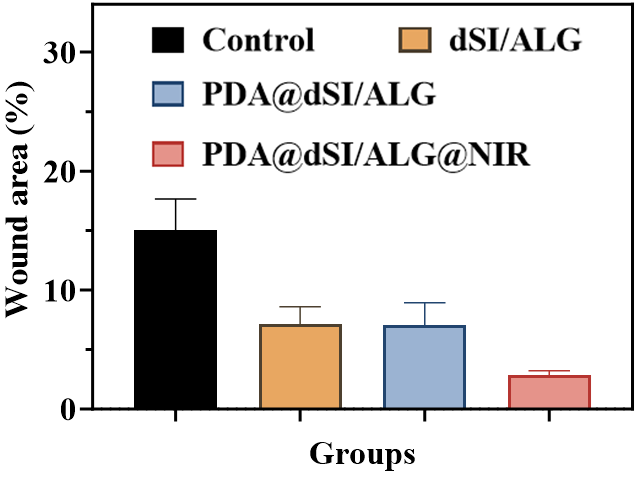


**Figure S8.** Quantitative assessment of wound area on day 9.
